# Catalytically Active Imine-based Covalent Organic Frameworks for Detoxification of Nerve Agent Simulants in Aqueous Media

**DOI:** 10.3390/ma12121974

**Published:** 2019-06-19

**Authors:** Sergio Royuela, Rodrigo Gil-San Millán, María J. Mancheño, M. Mar Ramos, José L. Segura, Jorge A. R. Navarro, Félix Zamora

**Affiliations:** 1 Departamento de Química Orgánica I, Facultad de CC. Químicas, Universidad Complutense de Madrid, 28040 Madrid, Spain; s.royuela@ucm.es (S.R.); mjmreal@quim.ucm.es (M.J.M.); 2 Departamento de Tecnología Química y Ambiental, Universidad Rey Juan Carlos, 28933 Madrid, Spain; mariamar.ramos@urjc.es; 3 Departamento de Química Inorgánica, Universidad de Granada, 18071 Granada, Spain; rodrigsm@correo.ugr.es; 4 Departamento de Inorgánica, Facultad de Ciencias, Universidad Autónoma de Madrid, 28049 Madrid, Spain; 5 Institute for Advanced Research in Chemical Sciences (IAdChem), Universidad Autónoma de Madrid, 28049 Madrid, Spain; 6 Condensed Matter Physics Center (IFIMAC), Universidad Autónoma de Madrid, 28049 Madrid, Spain; 7 Instituto Madrileño de Estudios Avanzados en Nanociencia (IMDEA-Nanociencia), Cantoblanco, 28049 Madrid, Spain

**Keywords:** covalent organic frameworks, catalysis, chemical warfare agents, detoxification

## Abstract

A series of imine-based covalent organic frameworks decorated in their cavities with different alkynyl, pyrrolidine, and *N*-methylpyrrolidine functional groups have been synthetized. These materials exhibit catalytic activity in aqueous media for the hydrolytic detoxification of nerve agents, as exemplified with nerve gas simulant diisopropylfluorophosphate (DIFP). These preliminary results suggest imine-based covalent organic frameworks (COFs) as promising materials for detoxification of highly toxic molecules.

## 1. Introduction

**Figure 1:**
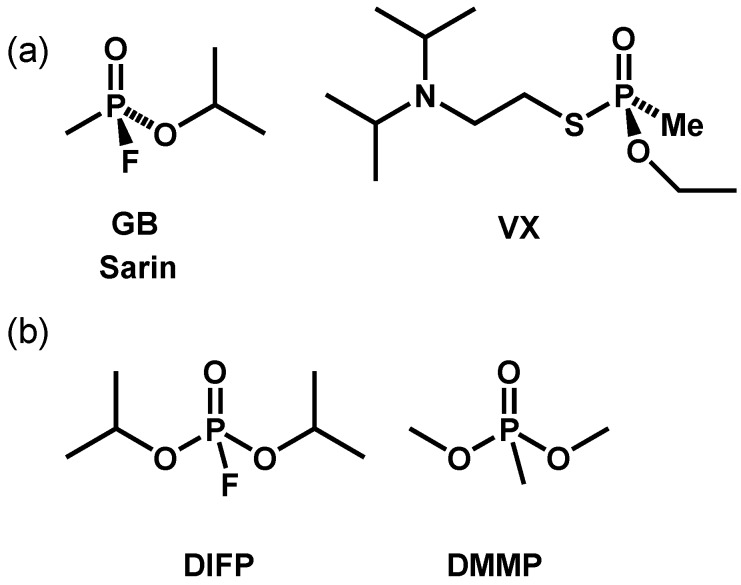
Examples of (**a**) highly toxic organophosphonate nerve agents and (**b**) nerve agent models of lower toxicity, used in conventional laboratories. DIFP: diisopropylfluorophosphate. DMMP: dimethylmethylphosphonate. GB: Sarin.

Nerve agents are amongst the most toxic chemical compounds known to mankind as a consequence of their easy penetration through human mucosa and ulterior damage of the central nervous system by the inhibition of acetylcholinesterase (AChE) [1]. Some examples of nerve agents include sarin (GB) and VX (Figure 1a). Although declared illegal by international agreements [2], recent attacks with chemical weapons against civil and military populations have been reported [3,4]. Consequently, protection and decontamination of these toxic chemicals is a very important societal challenge. Their hydrolytic degradation under environmental conditions (room temperature and ambient moisture) is one of the most convenient detoxification pathways, however, it will only take place in the presence of a suitable catalyst. Ideally, such catalyst should be a porous solid combining adsorptive and catalytic properties. The high toxicity of nerve agents prevents their use in standard research laboratories, with less toxic simulants (i.e., diisopropylfluorophosphate (DIFP), dimethylmethylphosphonate (DMMP)) (Figure 1b) being used in order to prove the suitability of a given material for decontamination purposes of the real nerve agents.

In recent years, covalent organic frameworks (COFs) have emerged as a new family of polymeric porous and crystalline materials based on the assembly of organic synthons by means of dynamic covalent bonds [5,6,7,8,9,10,11]. The modular nature of the COF architectures allows the fine tailoring of their structure and properties based on the selection of the molecular precursors [12]. Their intrinsic porosity is suited for applications related with gas adsorption and/or storage [5,7,9,10,13,14,15,16,17]. However, the incorporation of functional groups into their structures has envisioned different potential applications such as advanced materials for catalysis, solar energy collectors, optoelectronic devices, and clean energy applications [6,8,16,18,19,20,21]. Despite the fact that COFs show cavities that can be designed for several catalytic processes, still the number of reported examples is limited [22] and restricted mainly to classical carbon–carbon coupling reactions in organic solvents, such as Knoevenagel condensation [23], Michael addition [24,25], or Diels–Alder reaction [26]. In this context, the use of “click chemistry” is a highly efficient tool to functionalize COFs and decorate their cavities with specific molecules [27]. Noteworthy, this strategy allows control over the composition and density of functional groups being suitable for the incorporation of active catalytic sites [12].

By contrast, the field of catalysis in metal organic frameworks (MOFs) is currently much more developed as a consequence of the possibility of incorporating catalytic active sites both at the metal fragment and the organic linker [28,29,30]. In this regard, it has been demonstrated that the incorporation of nucleophilic sites in the structure of non-active MOFs gives rise to the hydrolytic degradation of ester bonds of DIFP nerve agent model in a 2-[tris(hydroxymethyl)-methylamino]-ethanesulfonic acid (TES)-buffered media [31]. Moreover, the incorporation of the naphthofluorescein pH sensor on a catalytically active MOF leads to the visual sensing of the easily hydrolysable diethylchlorophosphate [32].

**Scheme 1:**
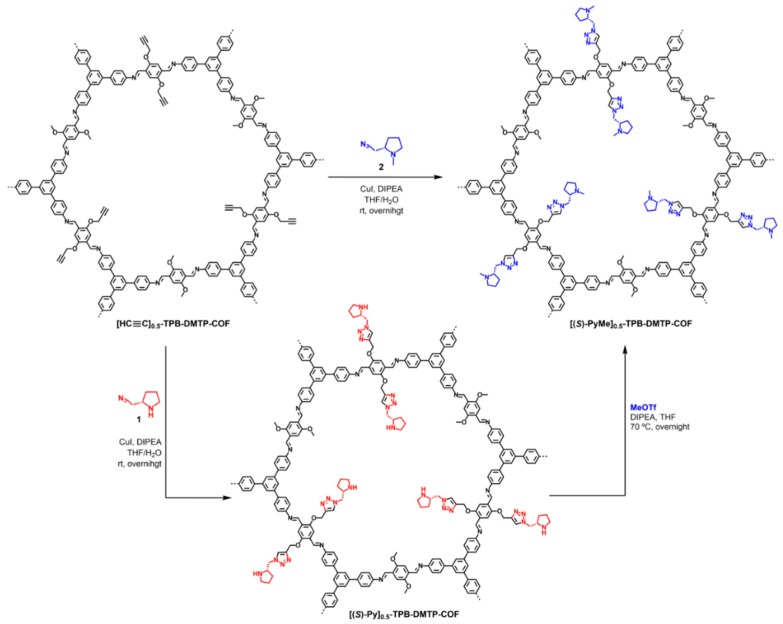
Synthesis of **[(*S*)-Py]_0.5_-TPB-DMTP-COF** and **[(*S*)-PyMe]_0.5_-TPB-DMTP-COF** via one-step or two-step postsynthetic modification.

In this work, we have considered that the porous framework structure and precise location of catalytically active sites in the porous structure of a COF can be useful for the introduction of nerve agent hydrolytic detoxification functions. Indeed, we demonstrate that an imine-based COF (Scheme 1) can be used for the hydrolytic detoxification of model nerve agent DIFP (Figure 1) in unbuffered aqueous media. Despite the relatively low hydrophilic behavior of COFs [33,34,35], DIFP hydrolysis takes place on the COF pore surface, representing one of the few cases of a catalytic reaction carried out in a COF cavity in water [25,36]. Moreover, we also report the functionalization of the pores with highly nucleophilic pyrrolidine residues, leading to a significant enhancement of the hydrolytic activity for the degradation of DIFP. We also show that the methylpyrrolidine moieties can be incorporated into the COF framework following two different postpolymerization functionalization strategies, leading to slightly different behavior (Scheme 1). In addition, we synthesized an analogous polymeric material with similar functionalities as in the COF, but with an amorphous nature. The comparison between the catalytic activity of the amorphous polymer and the covalent organic frameworks gives us a hint of the importance of pore accessibility and appropriate confinement effect of the substrate in the COF cavities.

## 2. Materials and Methods 

### 2.1. Characterization

All reactions with air sensitive materials were carried out under Ar using standard Schlenk techniques. Thin layer chromatography (TLC) was performed using pre-coated silica gel 60 F254 and developed in the indicated solvent system. Compounds were visualized under UV light (λ = 254 nm). Merck 60 (230–400 Mesh) silica gel was used for column chromatography. The following reagents were commercially available and were used as received: 2,5-dimethoxyterephthaldehyde (**DMTA**), CuI, *o*-DCB, *n*-butanol, NaN_3_ and *N*,*N*-diisopropylethylamine (DIPEA). 2,5-dihydroxyterephthaldehyde (**DHTA**) [25], 2,5-bis(prop-2-in-1-yloxy)terephthaldehyde (**BPTA**) [25], 1,3,5-tris-(4-aminophenyl)benzene (**TAPB**) [37], **[HC≡C]_0.5_-TPB-DMTP-COF** (TPB, triphenylbenzene; DMTP, dimethoxyterephthaldehyde) [25], **[(*S*)-Py]_0.5_-TPB-DMTP-COF** [25], (*S*)-2-(azidomethyl)pyrrolidine (**1**) [38], and (*S*)-2-(azidomethyl)-1-methylpyrrolidine (**2**) [39] were prepared according to reported procedures.

^1^H NMR and ^13^C NMR spectra were recorded on a Bruker AVIII-300 MHz (Billerica, MA, USA) spectrometer. Chemical shifts were reported in ppm and referenced to the residual non-deuterated solvent frequencies (CDCl_3_: δ 7.26 ppm for ^1^H, 77.0 ppm for ^13^C). Mass spectra were recorded by means of matrix-assisted laser desorption/ionization with a time-of-flight (MALDI-TOF) or fast atom bombardement (FAB) ionization techniques. Solids were analyzed by Fourier transform infrared (FTIR) spectroscopy on a Bruker TENSOR 27 (Billerica, MA, USA) on a diamond plate (attenuated total reflectance, ATR) or as films on sodium chloride. Powder X-ray diffraction (PXRD) measurements were carried out with X’PERT MPD (Malvern, UK), with conventional Bragg-Brentano geometry using Cu Kα (λ = 1.5406 Å) for values of 2θ from 2° to 40°. ^13^C CP/MAS NMR spectra were recorded on a Bruker AV-400 MHz Wide Bore (Billerica, MA, USA) spectrometer (probe: 4 mm MAS WB DVT). The sample rotation frequency was 12 kHz, and a 2.5 mm ZrO_2_ rotor was used. The N_2_ (77 K) and CO_2_ (273 K) adsorption isotherms were carried out on a Micromeritics Triflex equipment (Triflex, Micromeritics, Norcross, GA, USA). Prior to measurement, the materials were thermally activated at 393 K under dynamic vacuum (10^−5^ Pa). Thermogravimetric analysis was performed on a TGA-Q50 (TA Instruments, New Castle, DE, USA) instrument on a platinum plate, heating the samples under nitrogen atmosphere at a heating rate of 10 °C/min. Scanning electron microscopy (SEM) was carried out using a JOEL JSM 6335F (Tokyo, Japan) scanning electron microscope. The sample was dispersed over a slice of conductive adhesive (graphite) adhered to a flat copper platform sample holder and then coated with gold using a sputter coater before being submitted to SEM characterization.

### 2.2. Synthesis of COFs

**[HC≡C]_0.5_-TPB-DMTP-COF** [25]. Following the procedure previously described, a Pyrex vessel (Ø = 29 mm, h = 10 cm) was charged with **DMTA** (215 mg, 1.10 mmol), **BPTA** (268 mg, 1.11 mmol), TAPB (520 mg, 1.48 mmol), and *o*-DCB/*n*-Butanol/6M acetic acid (10 mL/10 mL/2.1 mL), degassed via three freeze-pump-thaw cycles, flame sealed, and kept at 130 °C for seven days. The precipitate was filtered, Soxhlet extracted with tetrahydrofuran (THF), and dried at 120 °C under vacuum, yielding 866.0 mg (94%) of a yellow powder. FTIR (ATR, cm^−1^): 3288, 2957, 2127, 1689, 1592, 1504, 1415, 1291, 1208, 1146, 1036, 878, 829, 694.

**[(*S*)-Py]_0.5_-TPB-DMTP-COF** [25]. To 157.1 mg of **[HC≡C]_0.5_-TPB-DMTP-COF** in 7 mL of a 3:1 THF/H_2_O mixture were added 43.9 mg (0.230 mmol) of CuI and 138 μL of *N,N*-diisopropylethylamine (DIPEA). The suspension was purged with argon for 5 min, and then 64.0 mg (0.507 mmol) of (*S*)-2-(azidomethyl)pyrrolidine (**1**) was added. The mixture was stirred overnight at room temperature under argon and centrifuged at 6000 rpm for 5 min. After washing with acetonitrile, H_2_O and THF, the solid was dried under vacuum at 120 °C, yielding an orange powder (215 mg, 97%). FTIR (ATR, cm^−1^): 2944, 1675, 1590, 1506, 1415, 1289, 1208, 1146, 1043, 830, 695.

**2-step-[(*S*)-PyMe]_0.5_-TPB-DMTP-COF.** 100 mg of **[(*S*)-Py]_0.5_-TPB-DMTP-COF** was suspended in anhydrous THF under argon together with 158 μL of DIPEA. Methyl triflate (47 μL) was subsequently added, and the mixture was stirred overnight at 70 °C. The solid was filtered, washed with 0.5 M NaOH, H_2_O, and THF, and dried under vacuum at 120 °C, yielding 109.0 mg of an orange solid. FTIR (ATR, cm^−1^): 2929, 2857, 1677, 1591, 1506, 1462, 1413, 1289, 1208, 1146, 1039, 830, 696.

**1-step-[(*S*)-Py]_0.5_-TPB-DMTP-COF.** To 107.3 mg of **[HC≡C]_0.5_-TPB-DMTP-COF** in 4.8 mL of a 3:1 THF/H_2_O mixture were added 30.7 mg (0.161 mmol) of CuI and 97 μL of DIPEA. The suspension was purged with argon for 5 min, and then 50.1 mg (0.357 mmol) of (*S*)-2-(azidomethyl)-1-methylpyrrolidine **(2)** was added. The mixture was stirred overnight at room temperature under argon and centrifuged at 6000 rpm for 5 min. After washing with acetonitrile, H_2_O and THF, the solid was dried under vacuum at 120 °C, yielding an orange powder (142 mg, 90%). FTIR (ATR, cm^-1^): 2941, 2800, 1670, 1591, 1507, 1415, 1291, 1208, 1146, 1041, 830, 696.

**[HC≡C]_0.5_-TPB-DMTP-Polym. DMTA** (29.3 mg, 0.151 mmol), **BPTA** (36.4 mg, 0.150 mmol), and **TAPB** (70.1 mg, 0.200 mmol) were dissolved in 15 mL of acetone. A few minutes after the addition of 1.5 mL of 6 M acetic acid, a yellow solid appeared. The suspension was stirred for an additional 2 h, filtered, and thoroughly washed with methanol and THF. The resulting solid was dried at 120 °C under vacuum to afford 86.5 mg (70%) of a yellow powder. FTIR (ATR, cm^−1^): 2962, 1678, 1605, 1512, 1411, 1283, 1205, 1187, 1033, 879, 825, 687.

**[(*S*)-PyMe]_0.5_-TPB-DMTP-Polym.** To a suspension of 75 mg of **[HC≡C]_0.5_-TPB-DMTP-Polym** in 3.5 mL of a 3:1 THF/H_2_O mixture were added 21.3 mg (0.112 mmol) of CuI and 69 μL of DIPEA. After purging the suspension with argon for 5 min, 30.1 mg (0.215 mmol) of (*S*)-2-(azidomethyl)-1-methylpyrrolidine (**2**) was added, and the mixture was stirred overnight at room temperature under argon. After centrifugation at 6000 rpm for 5 min, the solid was collected by filtration, washed with acetonitrile, H_2_O and THF, and dried under vacuum at 120 °C, yielding an orange powder (82.0 mg, 78%). FTIR (ATR, cm^−1^): 1679, 1609, 1512, 1412, 1286, 1207, 1186, 1036, 827, 698.

### 2.3. DIFP Heterogeneous Catalytic Degradation Tests

The degradation of DIFP was studied by employing 0.014 mmol of the repeating block structure of each COF suspended in 0.5 mL of bi-distilled H_2_O (unbuffered experiments) or 0.5 mL of 0.45 M *N*-ethylmorpholine buffered aqueous solution. Afterwards, 2.5 μL of DMSO (used as internal reference) and 2.5 μL (0.014 mmol) of DIFP were added to the suspension. The evolution of the concentration of DIFP was followed at room temperature by taking 0.2 μL aliquots of the supernatant solution, which were analyzed by gas chromatography (450 GC, Varian, Palo Alto, CA, USA). 

## 3. Results and Discussion

The syntheses of the COFs endowed with pyrrolidine moieties start with the synthesis of an alkynyl-functionalized COF (**[HC≡C]_0.5_-TPB-DMTP-COF**, Scheme 1) by following a procedure previously described [25,40]. The subsequent postsynthetic functionalization of **[HC≡C]_0.5_-TPB-DMTP-COF** was carried out in a two-step sequence. The first step involved a copper-catalyzed Huisgen’s 1,3-dipolar cycloaddition reaction [41,42] of the alkynyl-functionalized COF with a free-pyrrolidine- functionalized azide (**1**, Scheme 1) to afford a COF endowed with free pyrrolidine moieties. The second postsynthetic step involved the treatment of the free-pyrrolidine-functionalized COF with methyl triflate at 70 °C using THF as solvent to afford the corresponding COF endowed with methylated pyrrolidine moieties (**[(*S*)-PyMe]_0.5_-TPB-DMTP-COF**) with good yields (Scheme 1). 

**Figure 2:**
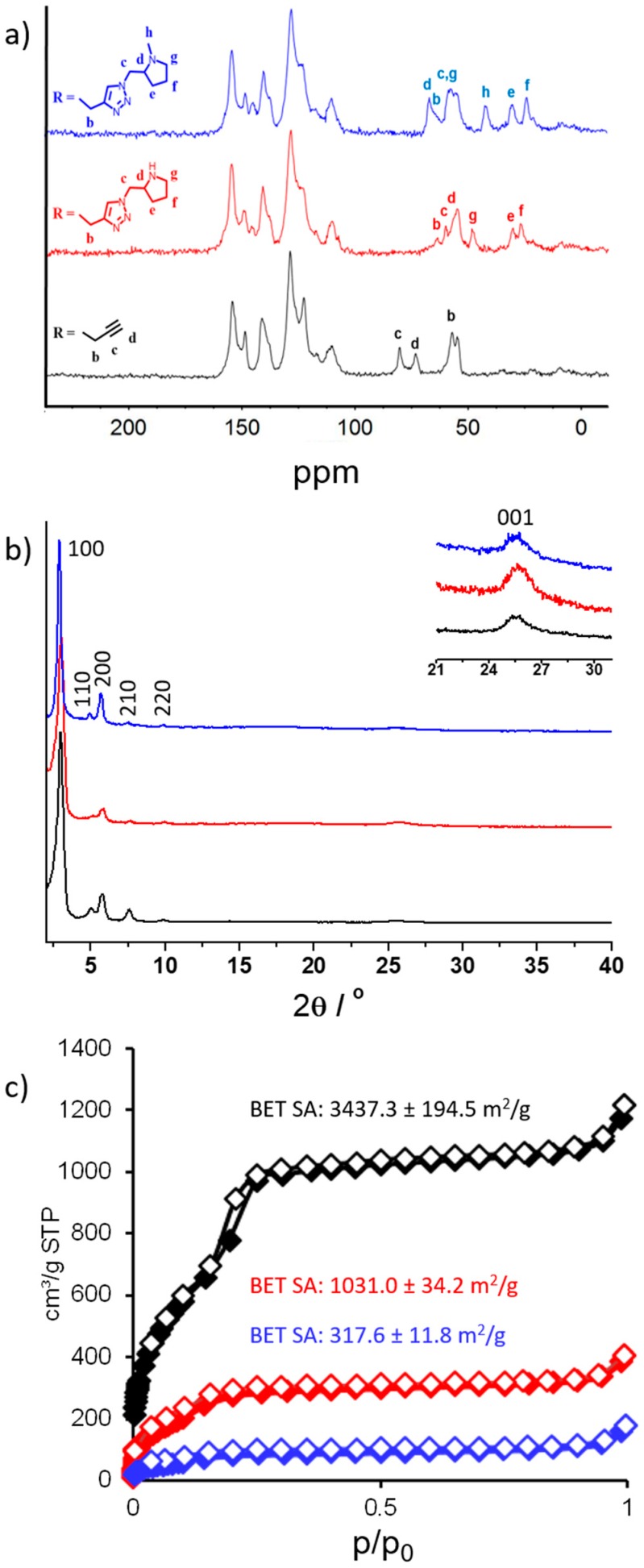
Comparative of (**a**) ^13^C CP/MAS NMR spectra, (**b**) PXRD patterns, and (**c**) N_2_ adsorption isotherms (77 K) for **[HC≡C]_0.5_-TPB-DMTP-COF** (black line), **[(*S*)-Py]_0.5_-TPB-DMTP-COF** (red line), and **1-step-[(*S*)-PyMe]_0.5_-TPB-DMTP-COF** (blue line). Open symbols correspond to the desorption branch.

Characterization of the COFs was carried out by Fourier transform infrared (FTIR) (Figures S1–S5), ^13^C cross-polarization magic-angle spinning NMR (^13^C CP/MAS NMR) spectroscopies (Figures S6–S9), and powder X-ray diffraction (PXRD) (Figure S10 and Table S1). FTIR and ^13^C CP/MAS NMR of **[HC≡C]_0.5_-TPB-DMTP-COF** and **[(*S*)-PyMe]_0.5_-TPB-DMTP-COF** show a similar profile (Figure 2a), which confirms that the structure of the COF skeleton was stable under the postsynthetic reaction conditions. By comparison of the aliphatic region, it was distinguished the incorporation of the methyl group by the deshielding of the signals corresponding to the -*C*H- and -*C*H_2_- next to the pyrrolidine nitrogen, which were shifted by 15 ppm, and the appearance of a new signal at 42 ppm corresponding to the N-*C*H_3_. PXRD of all COFs exhibited a similar diffraction pattern, corresponding to the AA stacking mode of a space group P6, corroborating their basic structural framework (Figure 2b and Table S1). PXRD of **[(*S*)-PyMe]_0.5_-TPB-DMTP-COF** also presented peaks related to those of the precursor COFs, indicating that the same lattice was maintained after functionalization.

The effect of the functional pendant groups on the material porosity was evaluated by N_2_ adsorption at 77 K (Figure 2c and Figure S11). The Brunauer–Emmett–Teller (BET) surface area, pore volume, and pore size distribution for **[HC≡C]_0.5_-TPB-DMTP-COF** and **[(*S*)-Py]_0.5_-TPB-DMTP-COF** were in good agreement with the previously reported data (Figure S12 and Table S2). In the case of **[(*S*)-PyMe]_0.5_-TPB-DMTP-COF,** the nitrogen sorption data were indicative that methylation is responsible for a significant decrease of pore accessibility to the N_2_ probe molecule (BET 95 m^2^ g^−1^ and pore volume of 0.108 cm^3^ g^−1^), although some degree of mesoporosity (NLDFT pore size of 2.9 nm) was maintained (Figure S13). In order to address this decrease of pore accessibility in the COF after the methylation reaction, we have carried out an alternative synthesis of **[(*S*)-PyMe]_0.5_-TPB-DMTP-COF** by using a one-step room-temperature post-synthetic functionalization of **[HC≡C]_0.5_-TPB-DMTP-COF**. With this aim, we have synthesized (*S*)-2-(azidomethyl)-1-methylpyrrolidine (**2**, Scheme 1) [39], in which the pyrrolidine moiety was already methylated. Subsequent copper-catalyzed Huisgen’s 1,3-dipolar cycloaddition reaction of **2** with **[HC≡C]_0.5_-TPB-DMTP-COF** afforded **[(*S*)-PyMe]_0.5_-TPB-DMTP-COF**. From now on, we will name this COF obtained with a one-step postsynthetic strategy **1-step-[(*S*)-PyMe]_0.5_-TPB-DMTP-COF,** while the analogue obtained via the two-steps post-synthetic strategy will be named **2-step-[(*S*)-PyMe]_0.5_-TPB-DMTP-COF**.

Fourier transform infrared (FTIR), ^13^C cross-polarization magic-angle spinning NMR (^13^C CP/MAS NMR) (Figure 2a) spectroscopies, and powder X-ray diffraction (PXRD) (Figure 2b) analysis showed identical patterns for **1-step-[(*S*)-PyMe]_0.5_-TPB-DMTP-COF** and **2-step-[(*S*)-PyMe]_0.5_-TPB-DMTP-COF,** which indicate that the networks remained identical after the postsynthetic steps. Nevertheless, a threefold increase in the BET surface area of **1-step-[(*S*)-PyMe]_0.5_-TPB-DMTP-COF** (317 m^2^ g^−1^) was observed in comparison with that determined with the parent **2-step-[(*S*)-PyMe]_0.5_-TPB-DMTP-COF** (95 m^2^ g^−1^). This difference in BET surfaces might be attributed to a partial further methylation of methylpyrrolidine residues to the corresponding dimethylpyrrolidonium triflate during the two-step synthetic process, which limits pore accessibility.

In order to have a further insight about the importance of pore accessibility in these materials, we have also addressed the synthesis of an amorphous polymer analogue to **[(*S*)-PyMe]_0.5_-TPB-DMTP-COFs**. Thus, an amorphous alkynyl-functionalized polymer was obtained by using the same monomers as those used for the synthesis of **[HC≡C]_0.5_-TPB-DMTP-COF,** but without the use of solvothermal conditions. The further reaction of this alkynyl-functionalized amorphous polymer with azide **2** afforded an amorphous nonporous polymer endowed with methylpyrrolidine moieties, **[(*S*)-PyMe]_0.5_-TPB-DMTP-Polym**. Characterization of this material by FTIR and ^13^C CP/MAS NMR presented no significant differences when compared with the analogue COFs, but it showed no diffraction peaks and its BET surface area was only 7.5 m^2^ g^−1^ (see ESI).

Finally, COFs were characterized by scanning electron microscopy (SEM), revealing that the morphology is conserved after the postsynthetic modification (Figures S14–S16). Thermal stability of all the frameworks was determined by thermogravimetric analysis (TGA). All the COFs were stable at temperatures above 250 °C, with the alkynyl-functionalized material (**[HC≡C]_0.5_-TPB-DMTP-COF**) being stable up to 400 °C, (Figures S17–S21).

**Figure 3:**
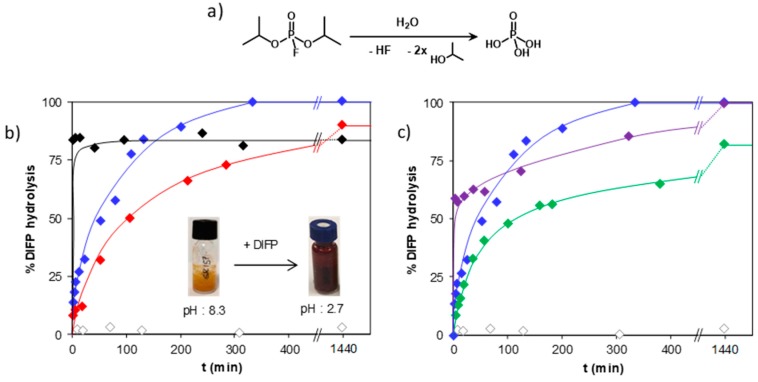
(**a**) Diisopropylfluorophosphate (DIFP) hydrolytic degradation reaction; (**b**) Reaction profiles for **[HC≡C]_0.5_-TPB-DMTP-COF** (black), **[(*S*)-Py]_0.5_-TPB-DMTP-COF** (red), and **1-step-[(*S*)-PyMe]_0.5_-TPB-DMTP-COF** (blue) in unbuffered aqueous media. The inset in b shows the change of color exhibited by **[HC≡C]_0.5_-TPB-DMTP-COF** concomitant to solution acidification after DIFP degradation; (**c**) Reaction profiles for **1-step-[(*S*)-PyMe]_0.5_-TPB-DMTP-COF** (blue), **2-step-[(*S*)-PyMe]_0.5_-TPB-DMTP-COF** (purple), and amorphous **[(*S*)-PyMe]_0.5_-TPB-DMTP-Polym** (green) in unbuffered aqueous media.

Once we fully characterized the synthetized materials, we proceeded to prove the possible utility of [HC≡C]_0.5_-TPB-DMTP-COF, [(*S*)-Py]_0.5_-TPB-DMTP-COF, 1-step-[(*S*)-PyMe]_0.5_-TPB-DMTP-COF, 2-step-[(*S*)-PyMe]_0.5_-TPB-DMTP-COF, and [(*S*)-PyMe]_0.5_-TPB-DMTP-Polym systems as heterogeneous catalysts of harmful molecules. With this aim, we have essayed the interaction of these systems with the nerve agent simulant diisopropylfluorophosphate (DIFP). The DIFP hydrolysis tests were performed in both unbuffered aqueous media and *N*-ethylmorpholine-buffered media (pH = 9.2) (Figure 3 and Table S3), according to previous reports of our group [43,44].

Noteworthy, **[HC≡C]_0.5_-TPB-DMTP-COF** exhibited a rather fast initial DIFP degradation rate (t_1/2_ < 2 min) in unbuffered aqueous media, which was accompanied with a concomitant color change from light orange to dark red (Figure 3 and Figure S22). This result can be attributed to the protonation of the imine residues as a consequence of the solution acidification during DIFP hydrolysis (pH change from initial 8.3 to 2.7). The system color change attributable to solution acidification was confirmed with the addition of different acids (i.e., acetic acid). In order to discard the possibility of this color change being due to partial decomposition of the COF, we stirred a suspension of 50 mg of COF in 25 mL of a sodium acetate buffer solution (pH 4.21) at room temperature for 24 h. The material exhibited a fast color change from yellow to red in this buffered medium. This color change was fully reversible after simple washing with methanol. The FITR and PXRD of the material isolated exhibited an identical profile without significant weight loss, indicating that the lattice remained unaltered after this treatment. Similar results have been previously observed for **[HC≡C]_0.5_-TPB-DMTP-COF** under harsher conditions (concentrated, 12 M HCl) [25]. The hydrolytic activity and color change are attributed to the Lewis basic nature of the imino residues constituting the framework. In this regard, an ethynyl synthon was observed to be nonactive in the DIFP degradation, suggesting that the catalytic activity of the COF materials is due to the nucleophilic nature of the imine groups.

However, despite the very fast degradation rate, the reaction was not completed because of the rapid poisoning of the catalyst, as a probable consequence of the protonation of the framework imine residues by DIFP acidic degradation products (HF, H_3_PO_4_). This limitation was circumvented with pyrrolidine and *N*-methylpyrrolidine functionalized COFs, namely **[(*S*)-Py]_0.5_-TPB-DMTP-COF**, **1-step-[(*S*)-PyMe]_0.5_-TPB-DMTP-COF,** and **2-step-[(*S*)-PyMe]_0.5_-TPB-DMTP-COF**, in which pyrrolidine nitrogen atoms acted as basic groups leading to the completion of DIFP degradation. As expected, the tertiary amine-doped COFs exhibited higher reaction rates than the secondary amine-doped COF because of their increased basicity. Indeed, the final pH after hydrolysis was 4.8 for the secondary amine, and above 5 for the COFs based on the tertiary amine. With regard to the amorphous **[(*S*)-PyMe]_0.5_-TPB-DMTP-Polym**, the final pH after a similar treatment was 4.2. Noteworthy, the catalytic activity of the crystalline porous **1-step-[(*S*)-PyMe]_0.5_-TPB-DMTP-COF** was higher than the related **2-step-[(*S*)-PyMe]_0.5_-TPB-DMTP-COF** and **[(*S*)-PyMe]_0.5_-TPB-DMTP-Polym** exhibiting lower pore accessibilities. It can be observed (Figure 3c) that the reaction was faster and reached completion at shorter times for the most nucleophilic and accessible **1-step-[(*S*)-PyMe]_0.5_-TPB-DMTP-COF** material. This behavior can be explained in terms of the confinement of the reactants in the cavity of the crystalline porous material, given that the chemical functionalities are similar in both materials. 

**Figure 4:**
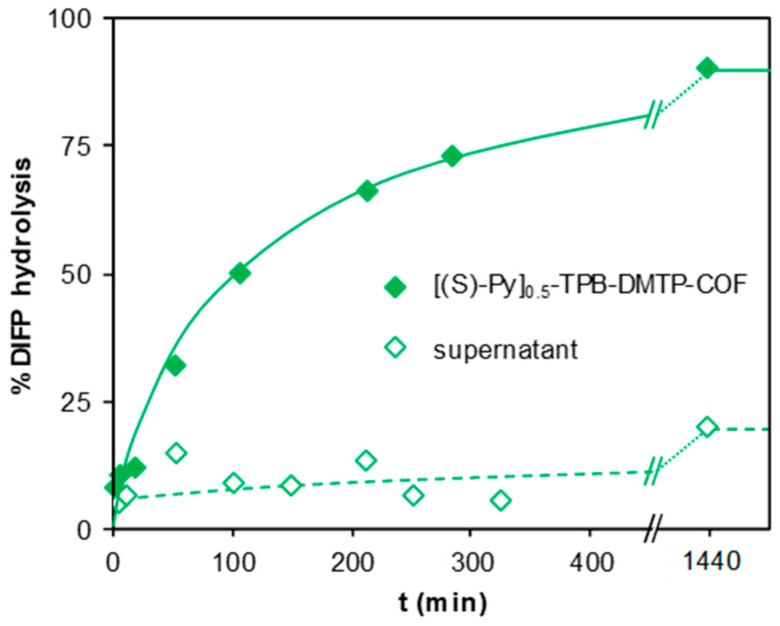
Filtration test to confirm the heterogeneous nature of the DIFP degradation process by **[(*S*)-Py]_0.5_-TPB-DMTP-COF** in unbuffered aqueous solution. DIFP degradation profile in the presence of **[(*S*)-Py]_0.5_-TPB-DMTP-COF** (solid diamonds) and supernatant solution after the filtration of the catalyst (open diamonds).

It is also worth pointing out that the catalytic process was heterogeneous since the filtration of the catalyst led to the halting of the degradation reaction, which is indicative of the heterogeneity of the DIFP degradation process (Figure 4).

Most of the detoxification studies of MOFs towards chemical warfare agents and their simulants are carried out in the presence of *N*-ethylmorpholine basic buffer (pH 9.2) [45]. The results show the hydrolytic degradation of DIFP by *N*-ethylmorpholine, which should be attributed to a combination of the nucleophilic and basic nature of the buffer system. In this regard, when the hydrolysis of DIFP was tested in the presence of a large excess of a standard buffer basic solution such as NaHCO_3_ (20 mg in 0.5 mL of H_2_O, pH = 8.04), we observed an initial fast hydrolysis, which was abruptly interrupted (pH = 7.80), so that the catalytic activity was lost upon solution acidification. Therefore, it can be concluded the benefit of the nucleophilic nature of the amino groups either in the homogeneous (*N*-ethylmorpholine) or in the heterogeneous phase (pyrrolidine-functionalized COFs). In this regard, catalytic studies of the **[(*S*)-Py]_0.5_-TPB-DMTP-COF** and **[(*S*)-PyMe]_0.5_-TPB-DMTP-COF** catalysts in the presence of *N*-ethylmorpholine were indicative of no further improvement of the catalytic activity (Figure S23).

## 4. Conclusions

Imine-based COFs are very promising materials for the detoxification of nerve agents in aqueous media as a consequence of the chemical robustness and nucleophilic nature of the imine bond [46,47]. Moreover, the incorporation of basic pyrrolidine and *N*-methylpyrrolidine functional groups gives rise to an increased heterogeneous detoxification activity of DIFP, being indicative of a synergistic effect of the combination of nucleophilicity and basicity of imine and pyrrolidine residues.

These preliminary results, together with the high chemical and thermal stability of imine-based COFs, suggest the potential of these materials as promising candidates towards the detoxification of highly toxic molecules. Moreover, the framework ordering and pore accessibility play an important role in the confinement of the reactants in the active catalytic sites in the COF materials.

Despite the relatively low hydrophilic behavior of a COF, this process represents the first catalytic reaction of COFs carried out in an aqueous medium.

These results suggest new opportunities for imine-based COF in catalytic processes beyond classical C–C coupling reactions carried out in organic solvents.

## Supplementary Materials

The following are available online at https://www.mdpi.com/1996-1944/12/12/1974/s1, Figure S1: FTIR (ATR) spectrum of **[HC≡C]_0.5_-TPB-DMTP-COF**, Figure S2: FTIR (ATR) spectrum of **[(*S*)-Py]_0.5_-TPB-DMTP-COF**, Figure S3: FTIR (ATR) spectrum of **2-step-[(*S*)-PyMe]_0.5_-TPB-DMTP-COF**, Figure S4: FTIR (ATR) spectrum of **2-step-[(*S*)-PyMe]_0.5_-TPB-DMTP-COF**, Figure S5: FTIR (ATR) spectrum of **[(*S*)-PyMe]_0.5_-TPB-DMTP-Polym**, Figure S6: ^13^C CP/MAS NMR of **[HC≡C]_0.5_-TPB-DMTP-COF**, Figure S7: ^13^C CP/MAS NMR of **[(*S*)-Py]_0.5_-TPB-DMTP-COF**, Figure S8: ^13^C CP/MAS NMR of **2-step-[(*S*)-PyMe]_0.5_-TPB-DMTP-COF**, Figure S9: ^13^C CP/MAS NMR of **1-step-[(*S*)-PyMe]_0.5_-TPB-DMTP-COF**, Figure S10: PXRD of **1-step-[(*S*)-PyMe]_0.5_-TPB-DMTP-COF** (blue), **2-step-[(*S*)-PyMe]_0.5_-TPB-DMTP-COF** (purple) and **[(*S*)-PyMe]_0.5_-TPB-DMTP-Polym** (green), Figure S11: N_2_ (77 K) adsorption isotherms for **1-step-[(*S*)-PyMe]_0.5_-TPB-DMTP-COF** (blue), **2-step-[(*S*)-PyMe]_0.5_-TPB-DMTP-COF** (purple) and **[(*S*)-PyMe]_0.5_-TPB-DMTP-Polym** (green), Figure S12: NLDFT pore size distribution for the essayed COF materials **[HC≡C]_0.5_-TPB-DMTP-COF** (a), **[(*S*)-Py]_0.5_-TPB-DMTP-COF** (b), **2-step-[(*S*)-PyMe]_0.5_-TPB-DMTP-COF** (c) and **1-step-[(*S*)-PyMe]_0.5_-TPB-DMTP-COF** (d), Figure S13: CO_2_ (298 K) adsorption isotherms for **[HC≡C]_0.5_-TPB-DMTP-COF** (black), **[(*S*)-Py]_0.5_-TPB-DMTP-COF** (red) and **[(*S*)-PyMe]_0.5_-TPB-DMTP-COF** (blue), Figure S14: SEM image of **[(*S*)-Py]_0.5_-TPB-DMTP-COF**, Figure S15: SEM image of **2-step-[(*S*)-PyMe]_0.5_-TPB-DMTP-COF**, Figure S16: SEM image of **1-step-[(*S*)-PyMe]_0.5_-TPB-DMTP-COF**, Figure S17: TGA profile of **[HC≡C]_0.5_-TPB-DMTP-COF**, Figure S18: TGA profile of **[(*S*)-Py]_0.5_-TPB-DMTP-COF**, Figure S19: TGA profile of **2-step-[(*S*)-PyMe]_0.5_-TPB-DMTP-COF**, Figure S20: TGA profile of **1-step-[(*S*)-PyMe]_0.5_-TPB-DMTP-COF**, Figure S21: TGA profile of **[(*S*)-PyMe]_0.5_-TPB-DMTP-Polym**, Figure S22: Effect of DIFP of addition to the essayed COF materials suspension on the pH and color in both buffer and unbuffered conditions, Figure S23: DIFP degradation studies in basic *N*-ethylmorpholine buffered media (pH = 9.2). Reaction profiles for **1-step-[(*S*)-PyMe]_0.5_-TPB-DMTP-COF** (blue) and **[(*S*)-PyMe]_0.5_-TPB-DMTP-Polym** (green), Table S1: Lattice parameters of the synthesized COFs, Table S2: Summary of textural properties of COF materials from N_2_ adsorption measurements at 77 K, Table S3: Catalytic degradation of diisopropylfluorophosphate (DIFP) nerve agent simulant.
