# Supplementary material for: Catalytically Active Imine-based Covalent Organic Frameworks for Detoxification of Nerve Agent Simulants in Aqueous Media
Source: Materials (Basel). 2019 Jun 19;12(12):1974. doi: 10.3390/ma12121974 (PMC6631658; doi:10.3390/ma12121974)
Supplement: Supplementary file 1 [file materials-12-01974-s001.pdf]

materials

Supplementary Materials

Catalytically Active Imine-based Covalent Organic Frameworks for Detoxification of Nerve Agent Simulants in Aqueous Media

Sergio Royuela [1,2], Rodrigo Gil-San Millán [3], María J. Mancheño [1], M. Mar Ramos [2], José L. Segura [1,*], Jorge A. R. Navarro [3,*] and Félix Zamora [4,5,6,7,*]

[1] Departamento de Química Orgánica I, Facultad de CC. Químicas, Universidad Complutense de Madrid, 28040 Madrid, Spain; s.royuela@ucm.es (S.R.); mjmreal@quim.ucm.es (M.J.M.);
[2] Departamento de Tecnología Química y Ambiental, Universidad Rey Juan Carlos, 28933 Madrid, Spain; mariamar.ramos@urjc.es
[3] Departamento de Química Inorgánica, Universidad de Granada, 18071 Granada, Spain; rodrigsm@correo.ugr.es
[4] Departamento de Inorgánica, Facultad de Ciencias, Universidad Autónoma de Madrid, 28049 Madrid, Spain
[5] Institute for Advanced Research in Chemical Sciences (IAdChem), Universidad Autónoma de Madrid, 28049 Madrid, Spain
[6] Condensed Matter Physics Center (IFIMAC), Universidad Autónoma de Madrid, 28049 Madrid, Spain
[7] Instituto Madrileño de Estudios Avanzados en Nanociencia (IMDEA-Nanociencia), Cantoblanco, E-28049 Madrid, Spain
* Correspondence: segura@quim.ucm.es (J.L.S.); jarn@ugr.es (J.A.R.N.); felix.zamora@uam.es (F.Z.)

FTIR spectra of COFs

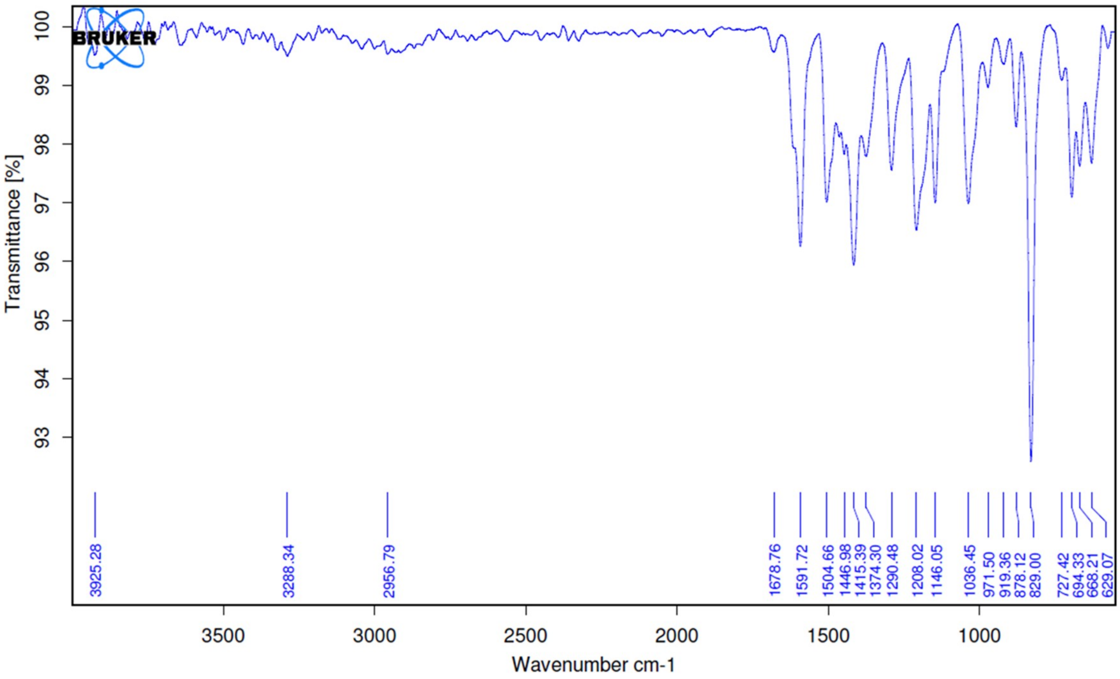

Figure S1. FTIR (ATR) spectrum of **[HC≡C]0.5-TPB-DMTP-COF**.

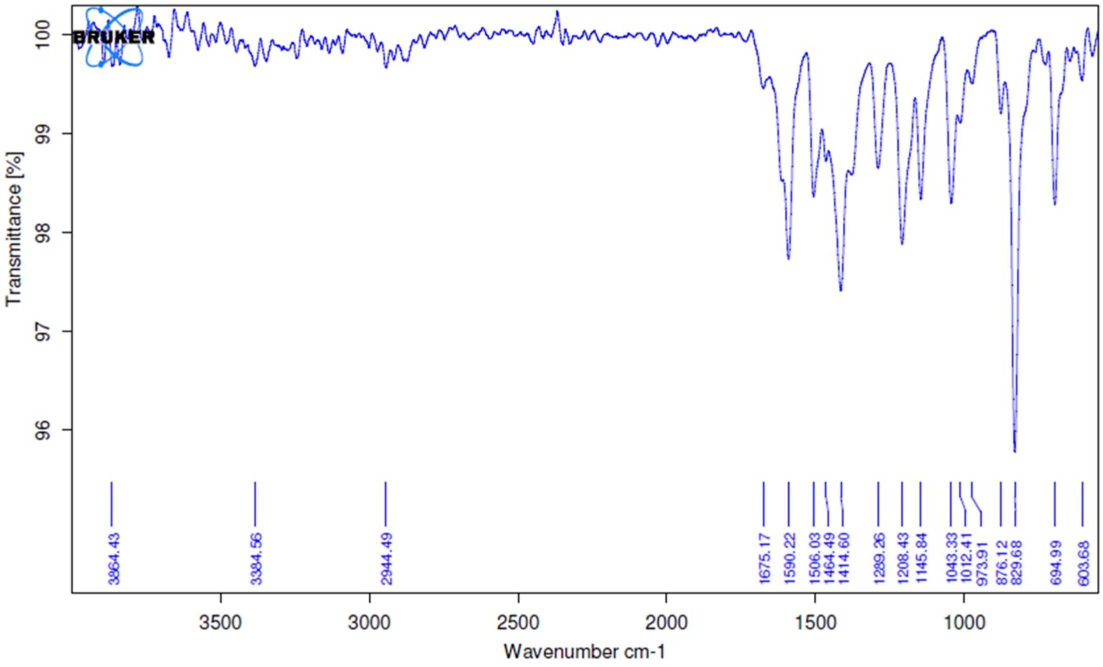

Figure S2. FTIR (ATR) spectrum of **[(*S*)-Py]0.5-TPB-DMTP-COF**.

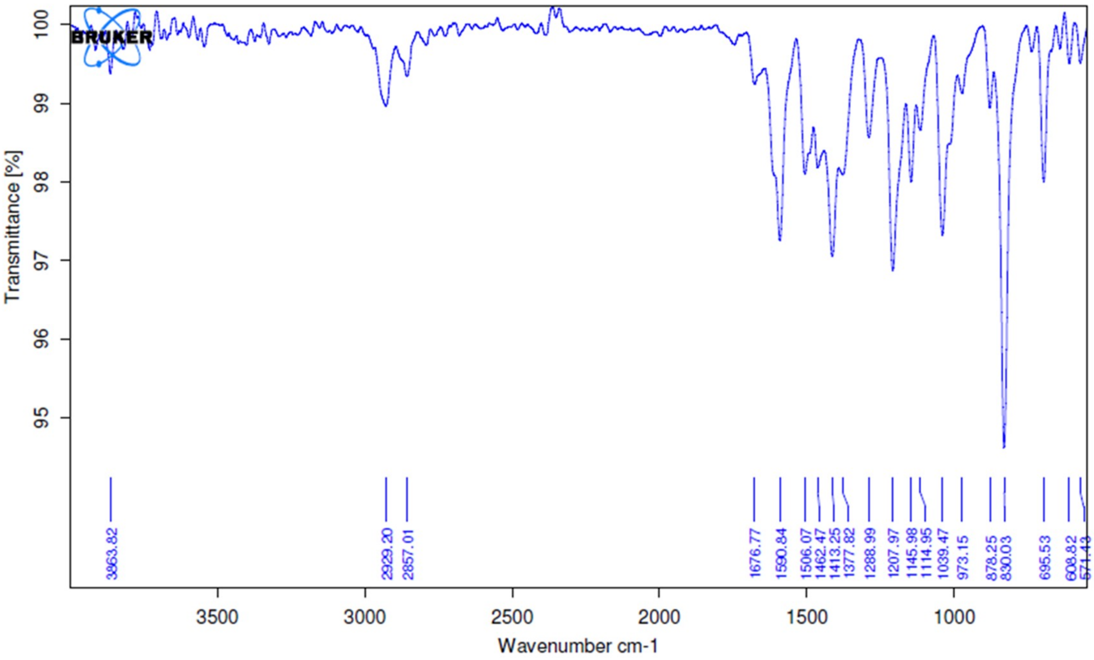

Figure S3. FTIR (ATR) spectrum of **2-step-[(*S*)-PyMe]$_{0.5}$-TPB-DMTP-COF**.

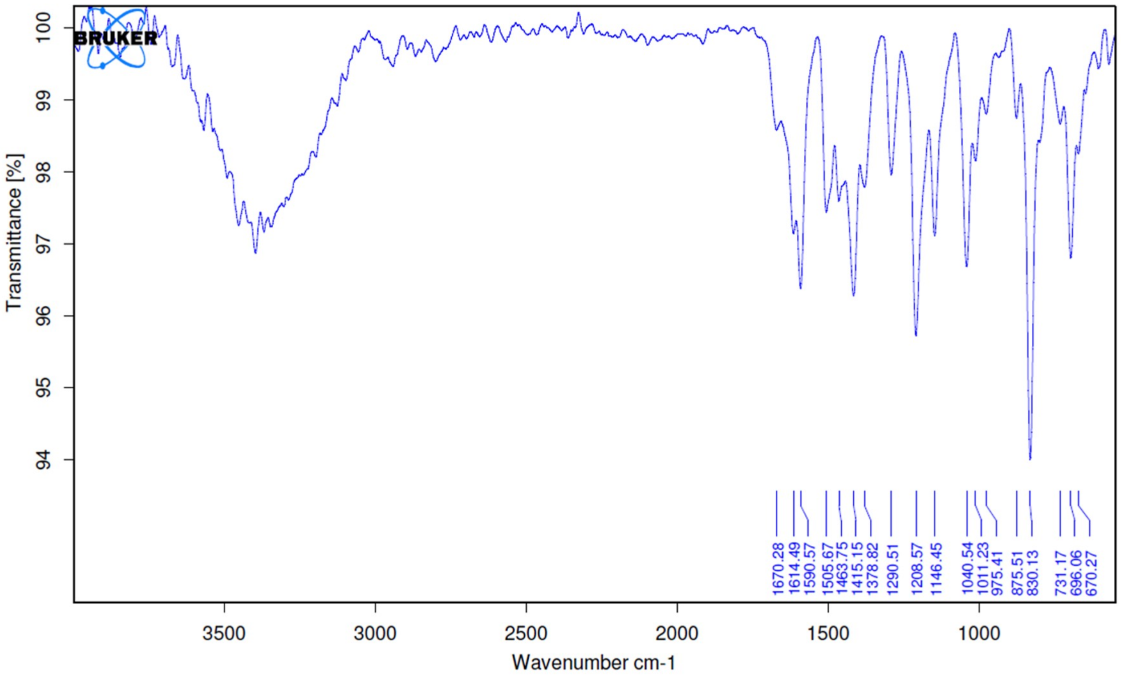

Figure S4. FTIR (ATR) spectrum of **2-step-[(*S*)-PyMe]$_{0.5}$-TPB-DMTP-COF**.

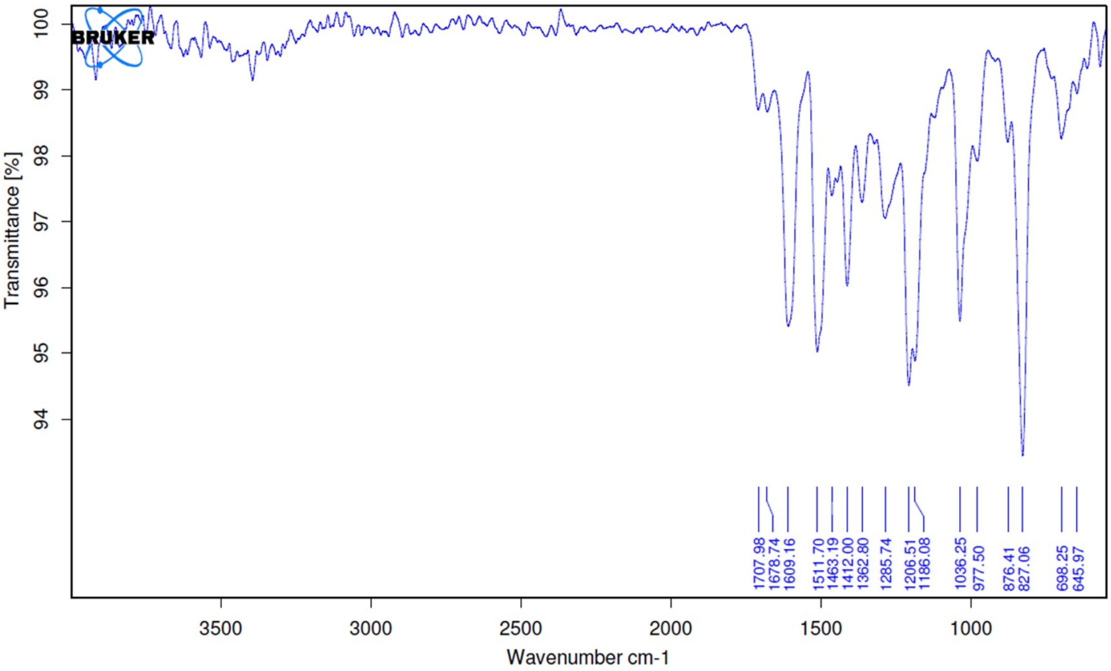

Figure S5. FTIR (ATR) spectrum of **[(*S*)-PyMe]$_{0.5}$-TPB-DMTP-Polym**.

- **^{13}C CP/MAS NMR spectra of COFs**

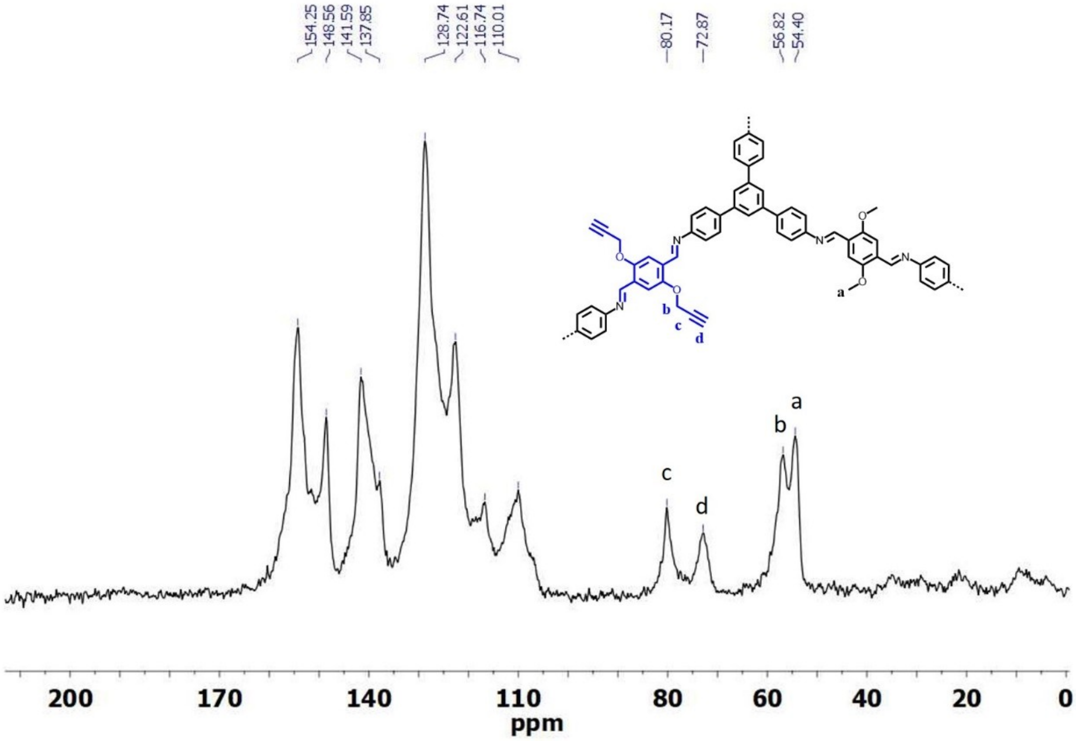

Figure S6. ^{13}C CP/MAS NMR of **[HC≡C]$_{0.5}$-TPB-DMTP-COF**.

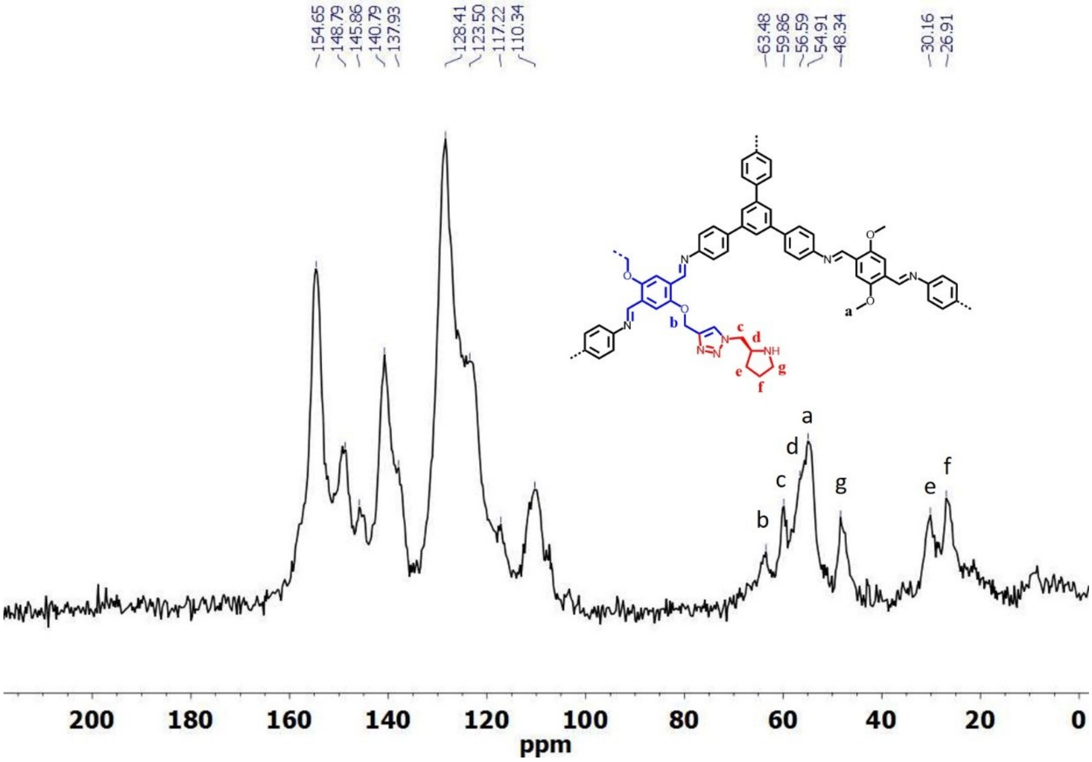

Figure S7. ^{13}C CP/MAS NMR of **[(*S*)-Py]0.5-TPB-DMTP-COF**.

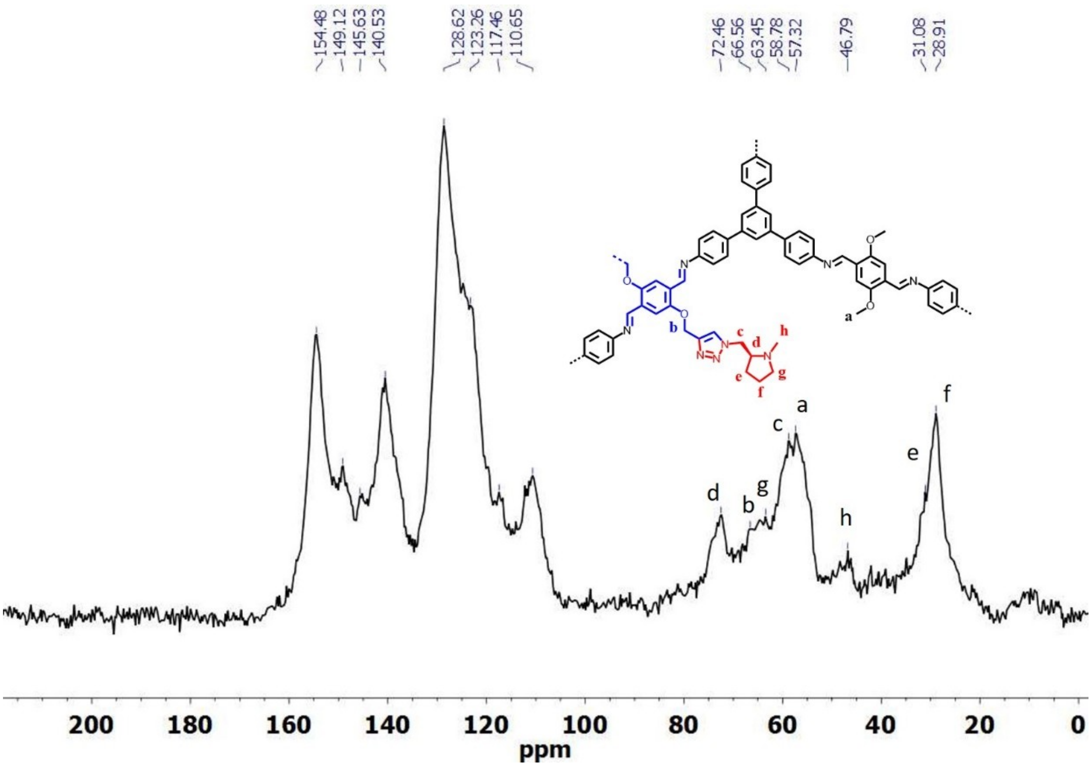

Figure S8. ^{13}C CP/MAS NMR of **2-step-[(*S*)-PyMe]0.5-TPB-DMTP-COF**.

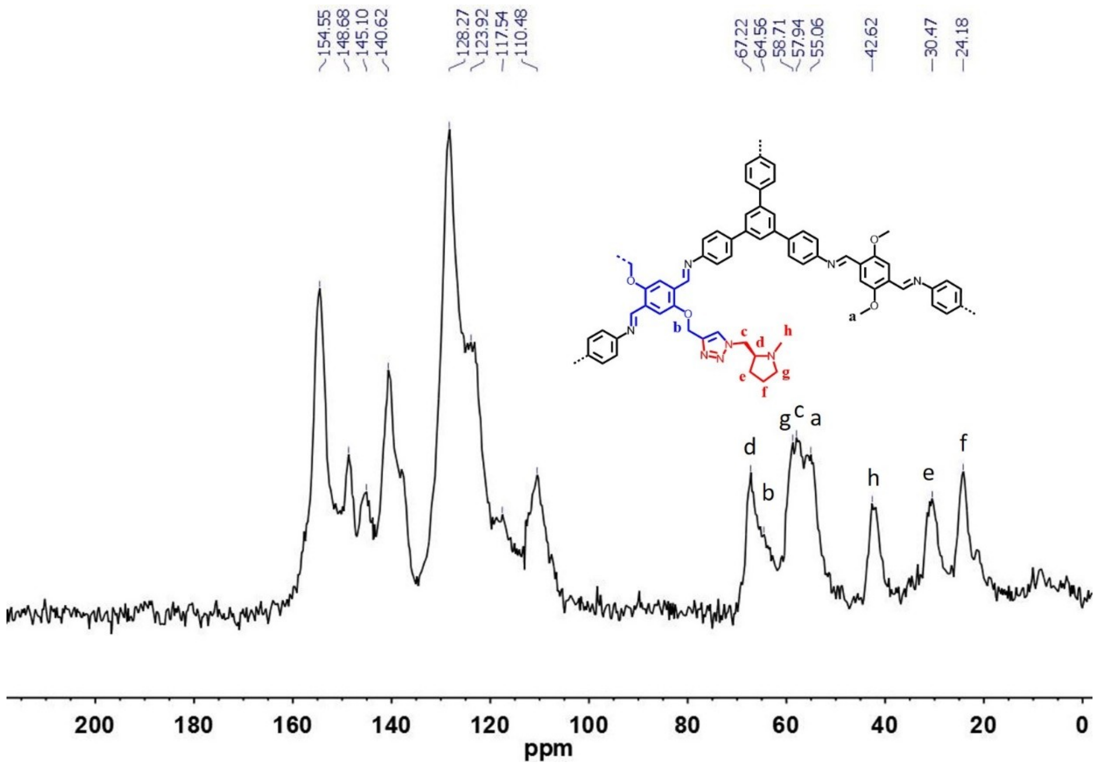

Figure S9. ^{13}C CP/MAS NMR of **1-step-[(*S*)-PyMe]$_{0.5}$-TPB-DMTP-COF**.

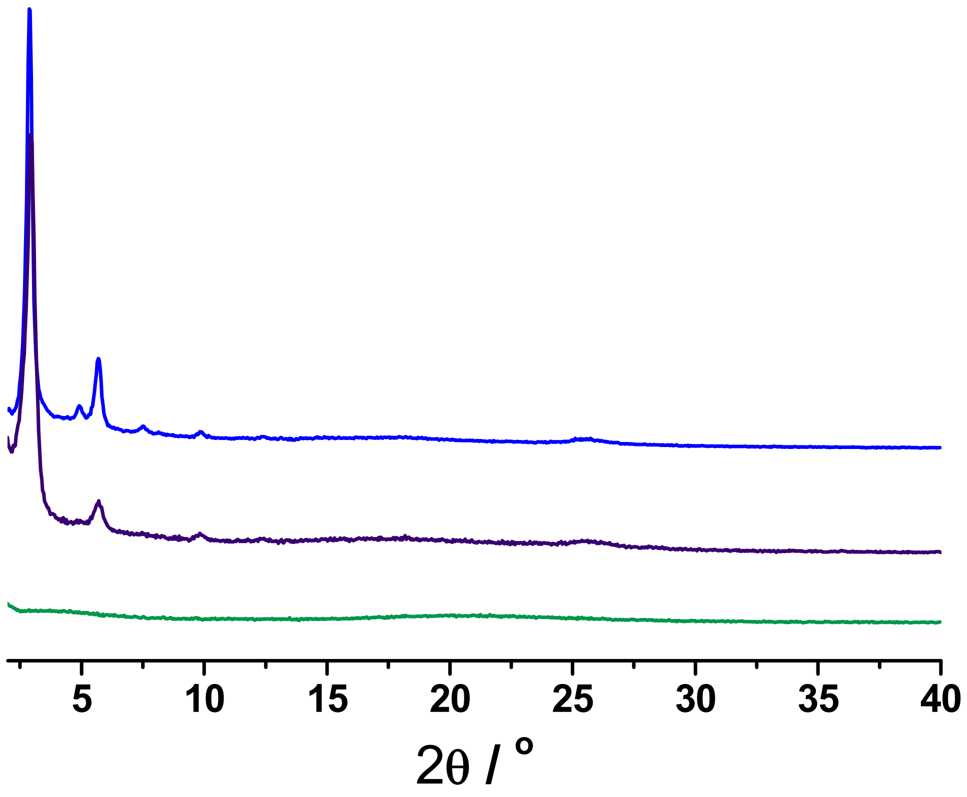

Figure S10. PXRD of **1-step-[(*S*)-PyMe]$_{0.5}$-TPB-DMTP-COF** (blue), **2-step-[(*S*)-PyMe]$_{0.5}$-TPB-DMTP-COF** (purple) and **[(*S*)-PyMe]$_{0.5}$-TPB-DMTP-Polym** (green).

Table S1. Lattice parameters of the synthesized COFs.

	**a = b (Å)**	**c (Å)**	**α = β (º)**	**γ (º)**
**[HC≡C]$_{0.5}$-TPB-DMTP-COF**	35.3	3.50	90	120
**[(*S*)-Py]$_{0.5}$-TPB-DMTP-COF**	34.7	3.51	90	120
**2-step-[(*S*)-PyMe]$_{0.5}$-TPB-DMTP-COF**	35.5	3.50	90	120
**1-step-[(*S*)-PyMe]$_{0.5}$-TPB-DMTP-COF**	35.7	3.50	90	120

- **Characterization of materials by N_2 (77 K) and CO_2 (273 K) adsorption**

Table S2. Summary of textural properties of COF materials from N_2 adsorption measurements at 77 K.

	BET surface area (m^2 g^{-1})	Pore volume (cm^3 g^{-1})	Pore size (nm)
**[HC≡C]$_{0.5}$-TPB-DMTP-COF**	3440	3.0	3.6
**[(*S*)-Py]$_{0.5}$-TPB-DMTP-COF**	1030	0.40	3.3
**2-step-[(*S*)-PyMe]$_{0.5}$-TPB-DMTP-COF**	95	0.06	2.9
**1-step-[(*S*)-PyMe]$_{0.5}$-TPB-DMTP-COF**	318	0.20	3.0
**[(*S*)-PyMe]$_{0.5}$-TPB-DMTP-Polym**	7.5	-	-

N_2 adsorption isotherms

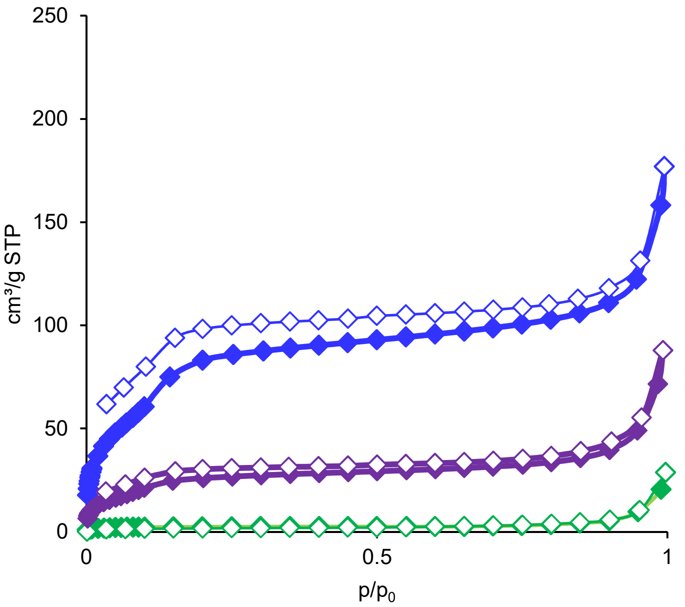

Figure S11. N_2 (77 K) adsorption isotherms for **1-step-[(*S*)-PyMe]$_{0.5}$-TPB-DMTP-COF** (blue), **2-step-[(*S*)-PyMe]$_{0.5}$-TPB-DMTP-COF** (purple) and **[(*S*)-PyMe]$_{0.5}$-TPB-DMTP-Polym** (green).

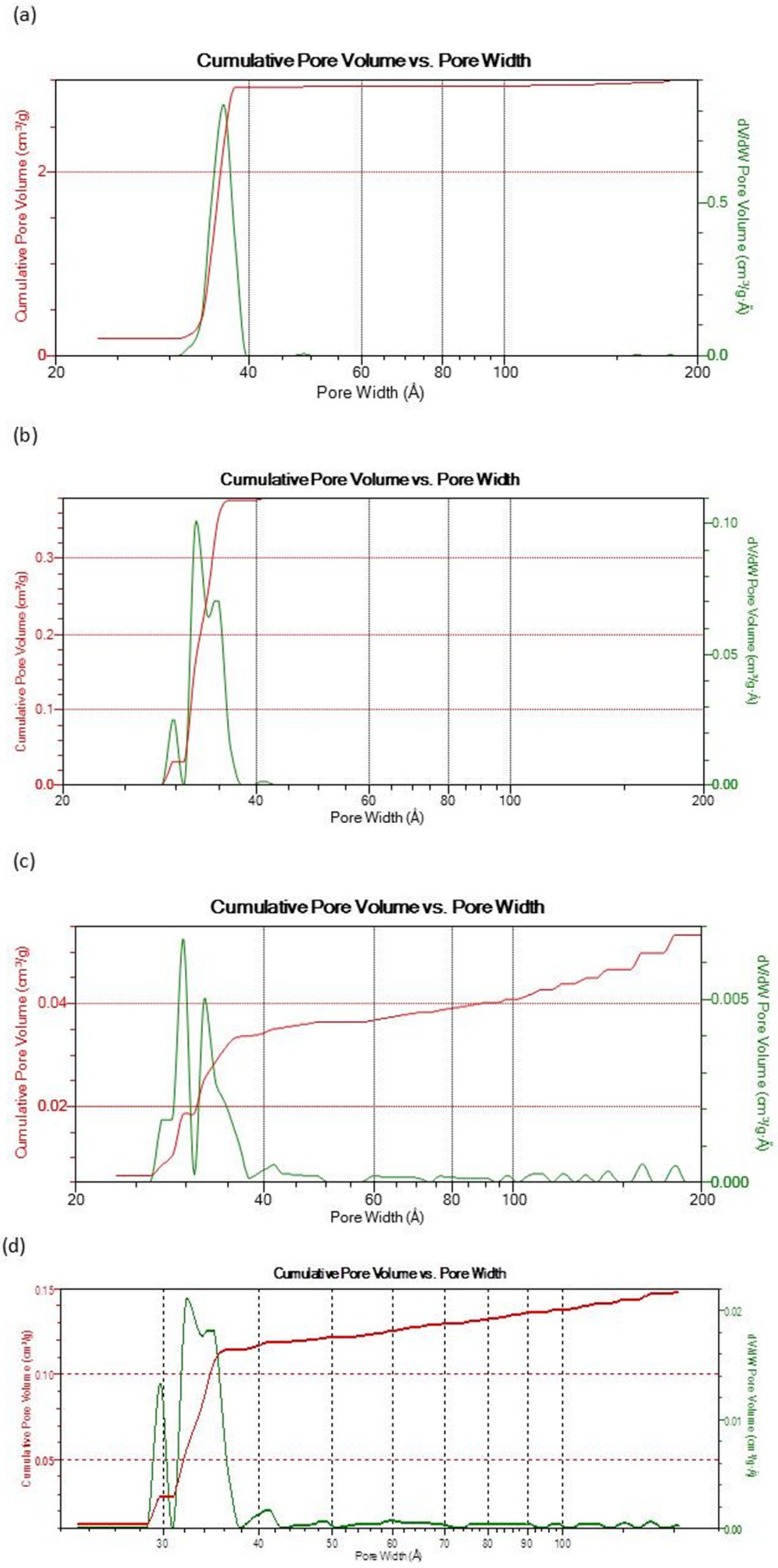

Figure S12. NLDFT pore size distribution for the essayed COF materials **[HC≡C]$_{0.5}$-TPB-DMTP-COF** (a), **[(*S*)-Py]$_{0.5}$-TPB-DMTP-COF** (b), **2-step-[(*S*)-PyMe]$_{0.5}$-TPB-DMTP-COF** (c) and **1-step-[(*S*)-PyMe]$_{0.5}$-TPB-DMTP-COF** (d).

CO_2 adsorption isotherms

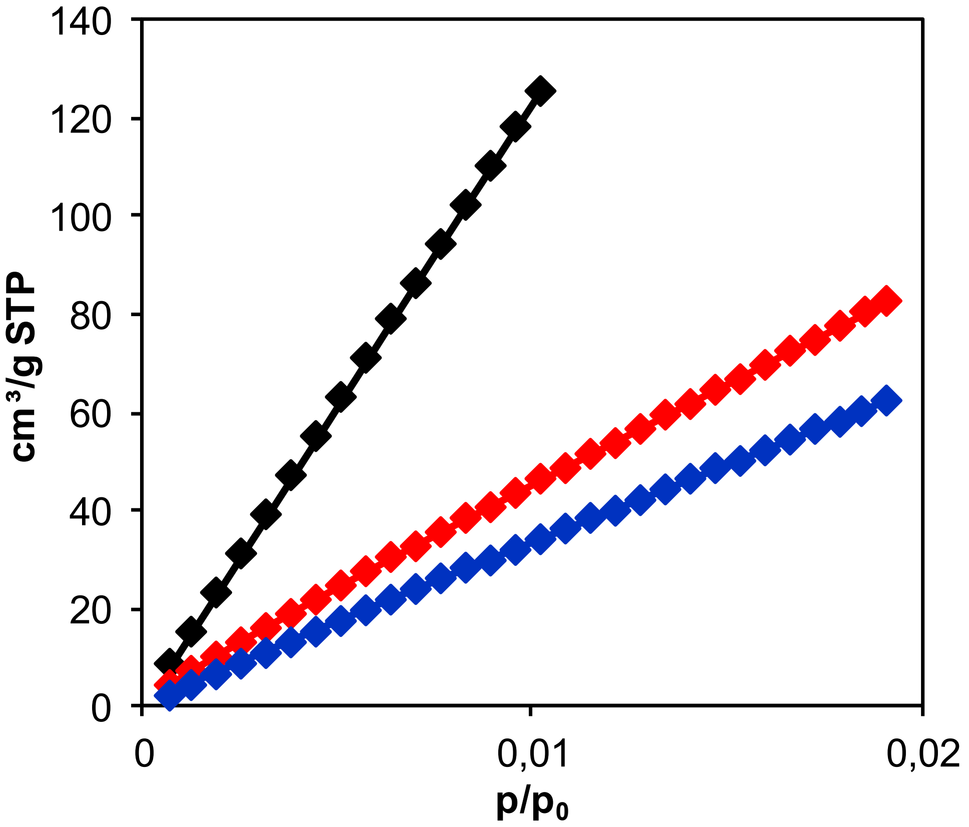

Figure S13. CO_2 (298 K) adsorption isotherms for **[HC≡C]$_{0.5}$-TPB-DMTP-COF** (black), **[(*S*)-Py]$_{0.5}$-TPB-DMTP-COF** (red) and **[(*S*)-PyMe]$_{0.5}$-TPB-DMTP-COF** (blue).

- **SEM images**

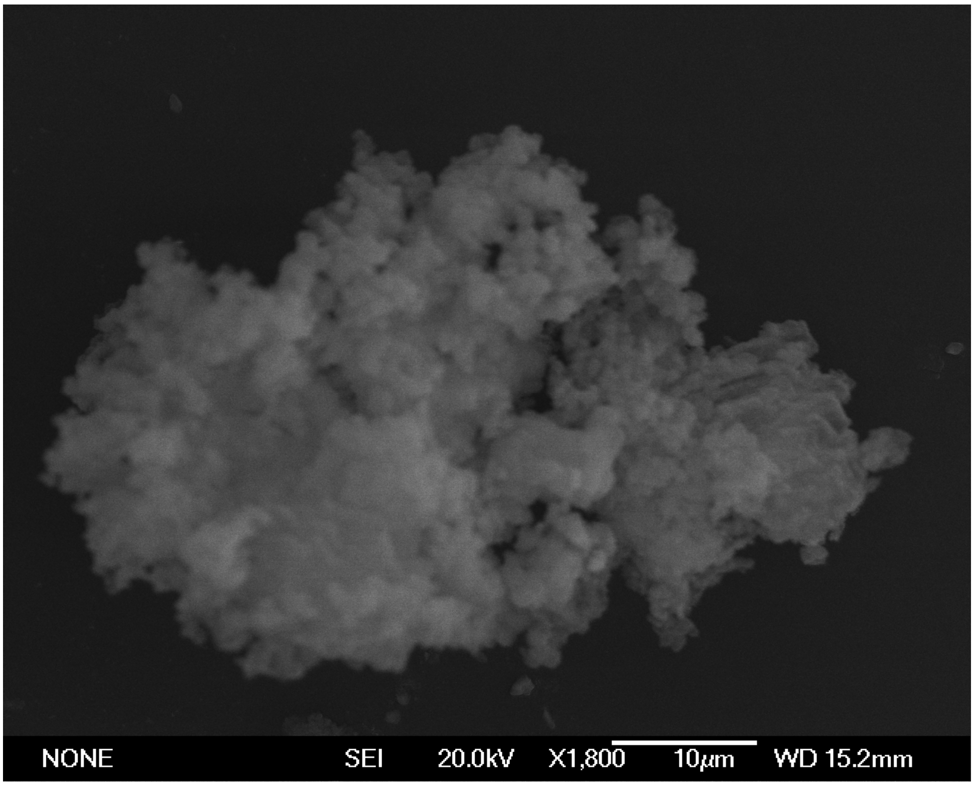

Figure S14. SEM image of **[(*S*)-Py]$_{0.5}$-TPB-DMTP-COF**.

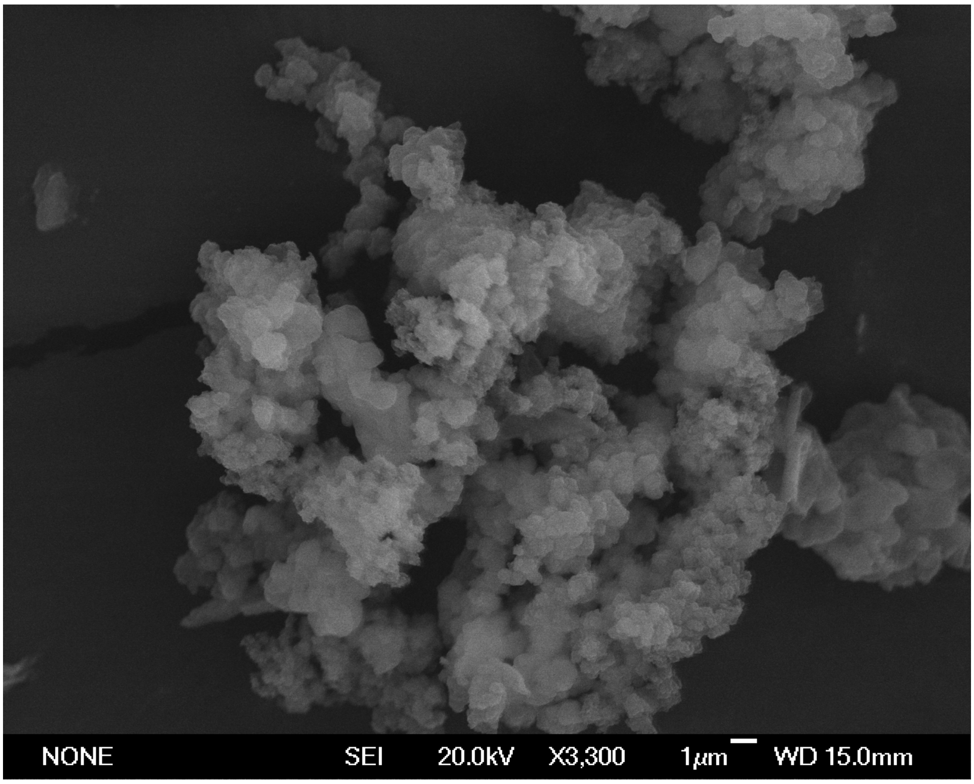

Figure S15. SEM image of **2-step-[(*S*)-PyMe]$_{0.5}$-TPB-DMTP-COF**.

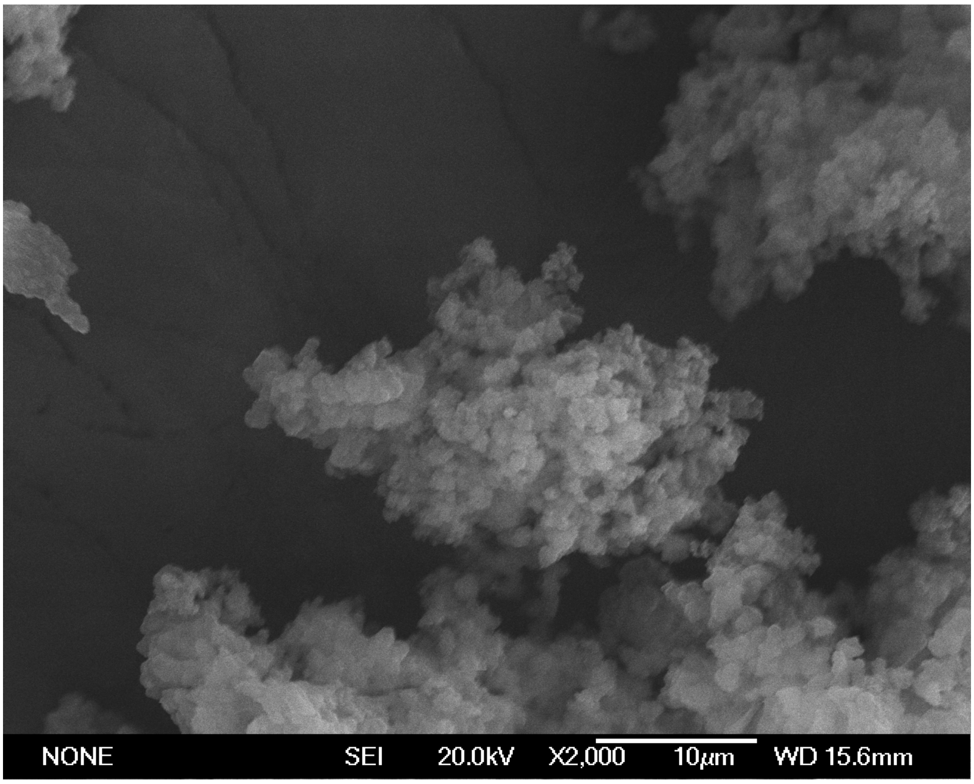

Figure S16. SEM image of **1-step-[(*S*)-PyMe]$_{0.5}$-TPB-DMTP-COF**.

- **TGA of COFs**

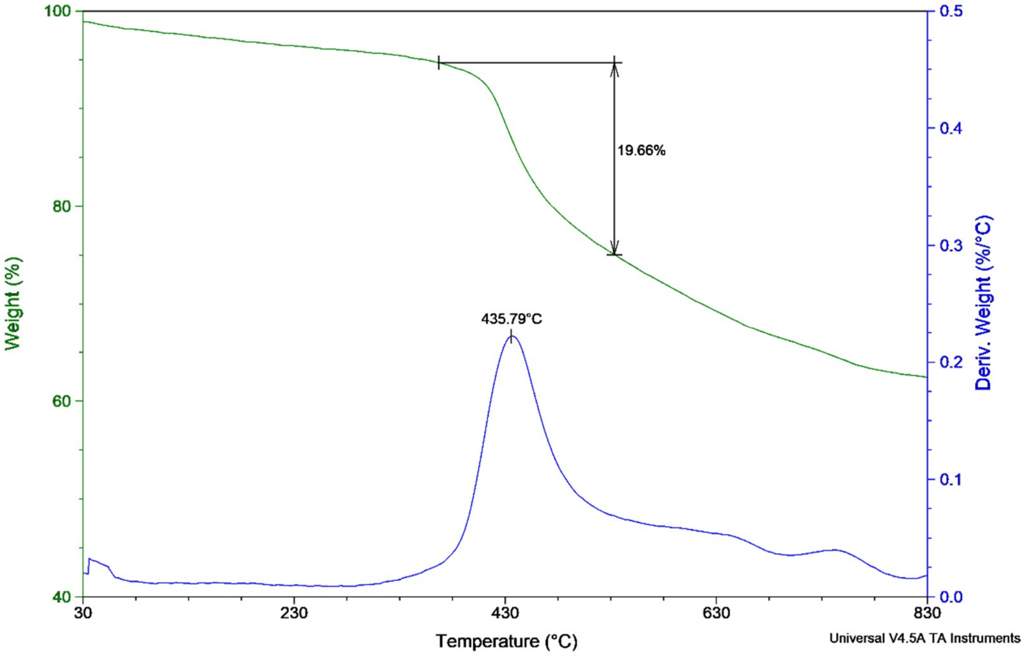

Figure S17. TGA profile of **[HC≡C]$_{0.5}$-TPB-DMTP-COF**.

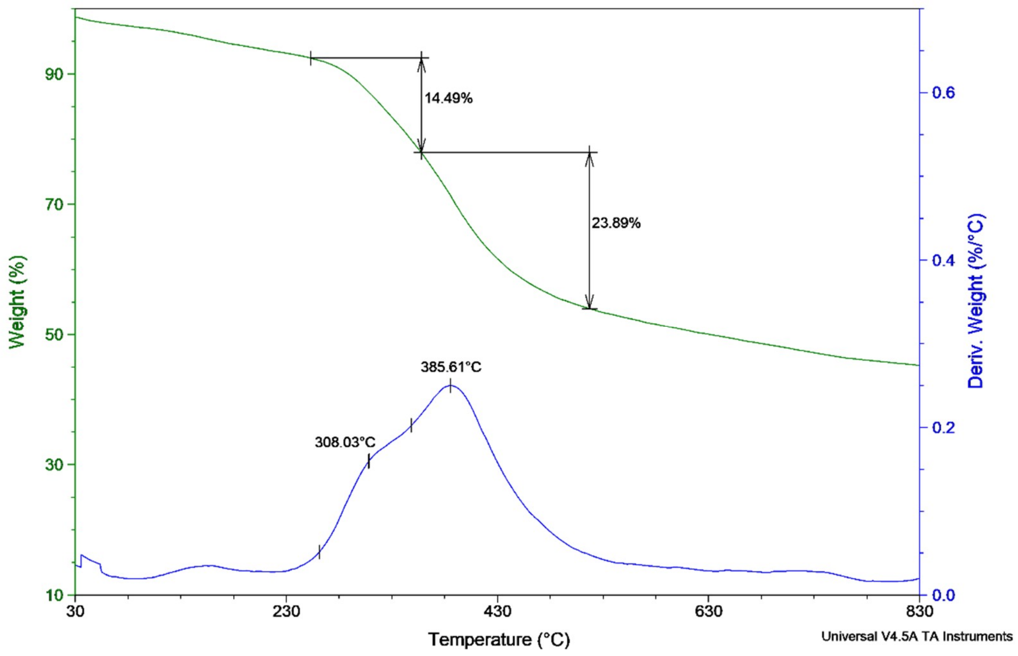

Figure S18. TGA profile of **[(*S*)-Py]$_{0.5}$-TPB-DMTP-COF**.

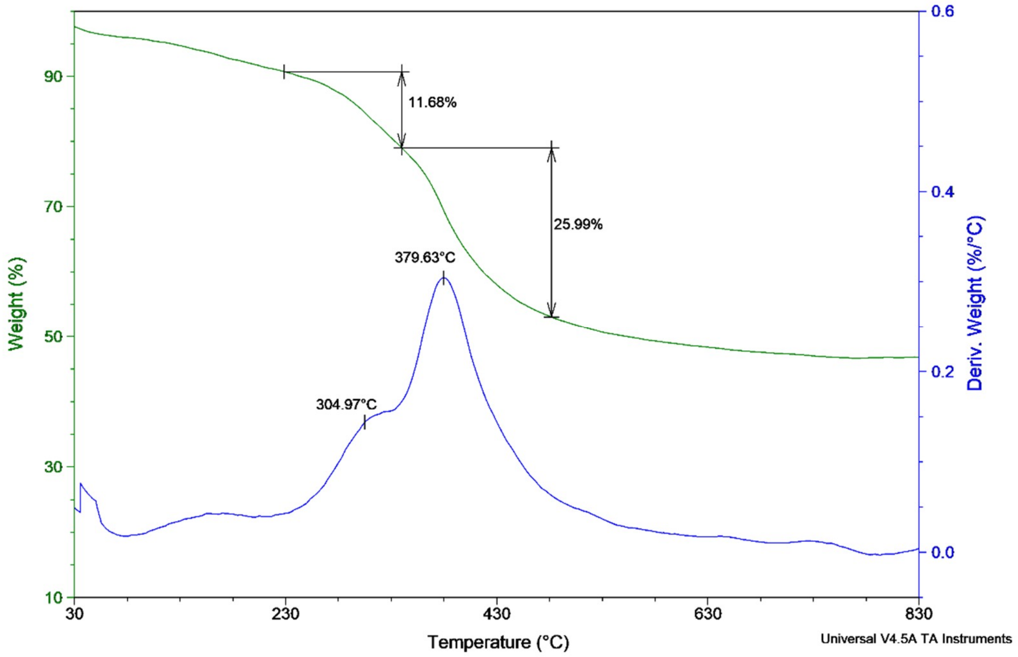

Figure S19. TGA profile of **2-step-[(*S*)-PyMe]$_{0.5}$-TPB-DMTP-COF**.

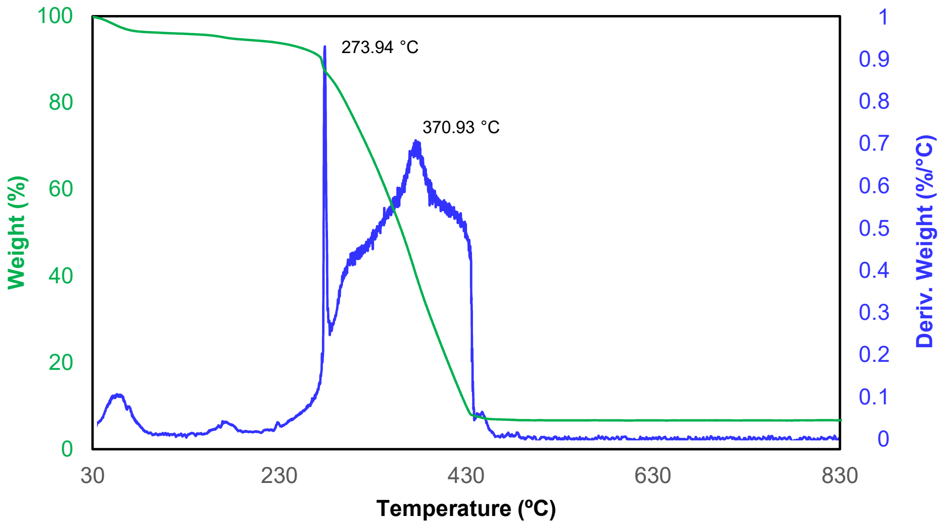

Figure S20. TGA profile of **1-step-[(*S*)-PyMe]$_{0.5}$-TPB-DMTP-COF**.

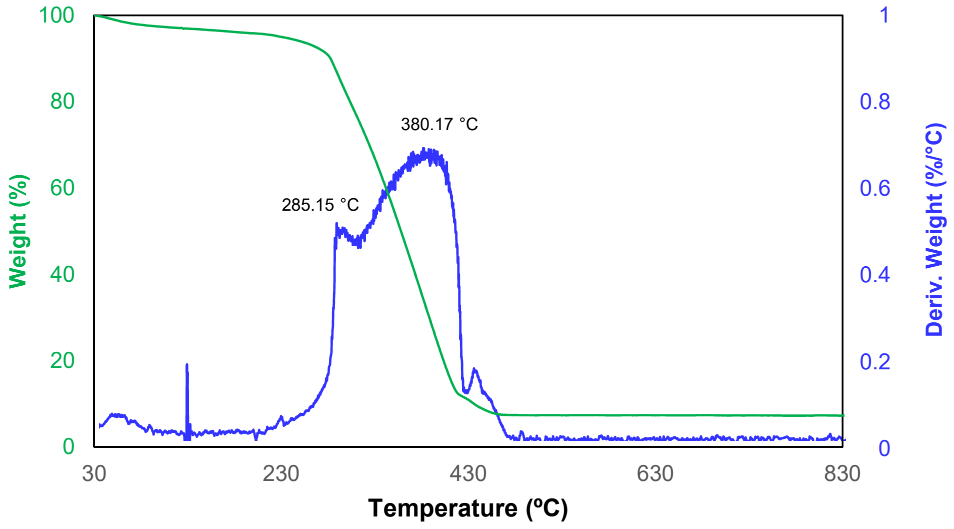

Figure S21. TGA profile of **[(*S*)-PyMe]$_{0.5}$-TPB-DMTP-Polym**.

- **Elemental Analysis**

- **[HC≡C]$_{0.5}$-TPB-DMTP-COF**

  Calculated - C: 80.75 %, H: 4.84 %, N: 6.73 %
  Experimental - C: 78.73 %, H: 5.06 %, N: 6.46 %
  Experimental - C: 79.20 %, H: 4.99 %, N: 6.48 %
  Experimental - C: 79.04 %, H: 5.05 %, N: 6.47 %

- **[(*S*)-Py]$_{0.5}$-TPB-DMTP-COF**

  Calculated - C: 73.04 %, H: 5.57 %, N: 15.49 %
  Experimental - C: 69.47 %, H: 5.48 %, N: 13.75 %
  Experimental - C: 69.93 %, H: 5.52 %, N: 13.81 %
  Experimental - C: 69.65 %, H: 5.53 %, N: 13.80 %

- **2-step-[(*S*)-PyMe]$_{0.5}$-TPB-DMTP-COF**

  Calculated - C: 73.36 %, H: 5.79 %, N: 15.10 %
  Experimental - C: 70.11 %, H: 5.50 %, N: 13.17 %
  Experimental - C: 69.69 %, H: 5.40 %, N: 13.17 %
  Experimental - C: 70.41 %, H: 5.47 %, N: 13.21 %

- **1-step-[(*S*)-PyMe]$_{0.5}$-TPB-DMTP-COF**

  Calculated - C: 73.36 %, H: 5.79 %, N: 15.10 %
  Experimental - C: 70.38 %, H: 5.64 %, N: 13.12 %
  Experimental - C: 70.47 %, H: 5.52 %, N: 13.08 %

- **Catalysis heterogeneity test**

Table S3. Catalytic degradation of diisopropylfluorophosphate (DIFP) nerve agent simulant.

	No buffer	Buffer
**[HC≡C]$_{0.5}$-TPB-DMTP-COF**	83.8	100
**[(*S*)-Py]$_{0.5}$-TPB-DMTP-COF**	89.4	100
**2-step-[(*S*)-PyMe]$_{0.5}$-TPB-DMTP-COF**	99.0	100
**1-step-[(*S*)-PyMe]$_{0.5}$-TPB-DMTP-COF**	100	100
**[(*S*)-PyMe]$_{0.5}$-TPB-DMTP-Polym**	84.1	100

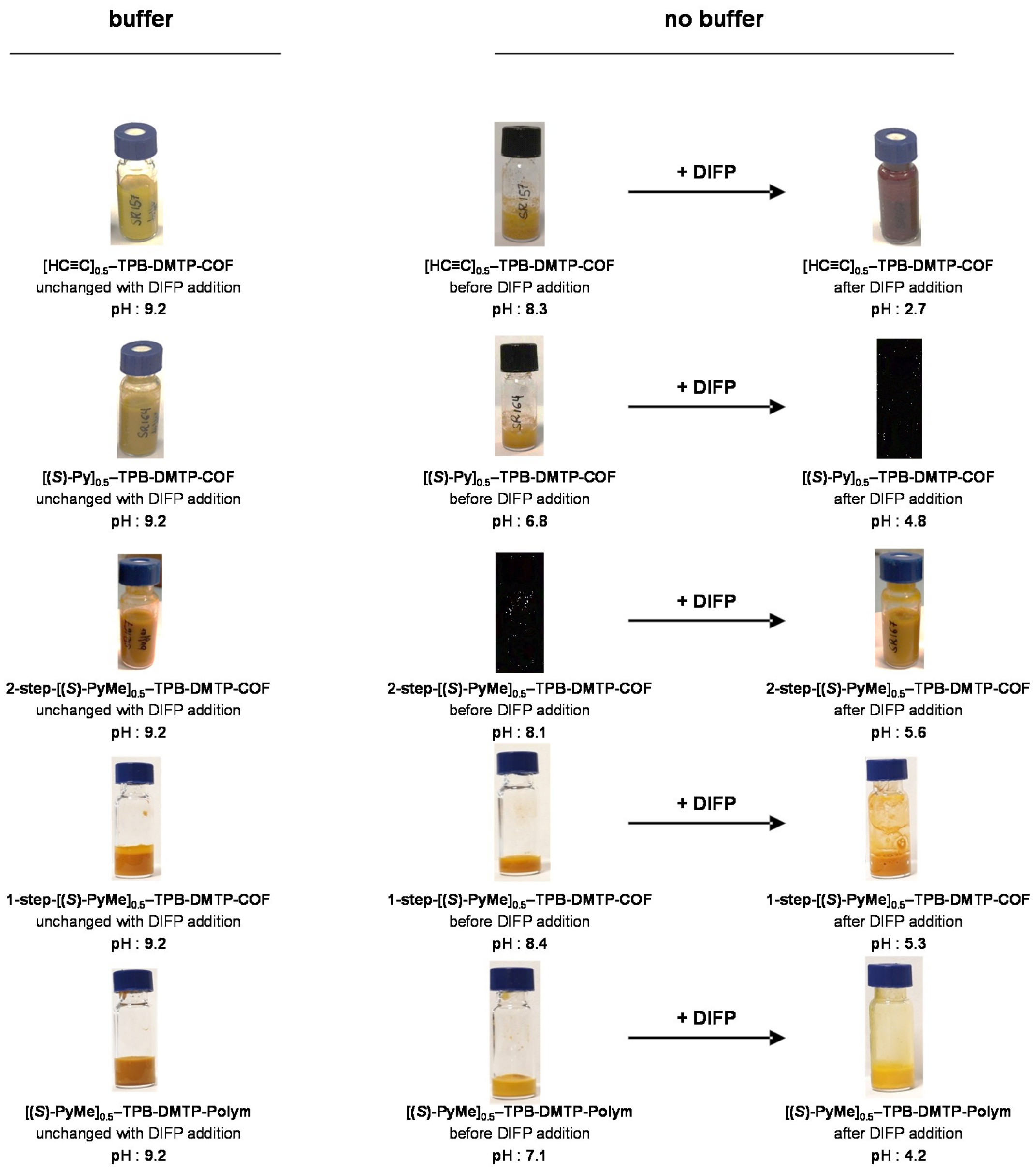

Figure S22. Effect of DIFP of addition to the essayed COF materials suspension on the pH and color in both buffer and unbuffered conditions.

- **Catalysis in buffered media**

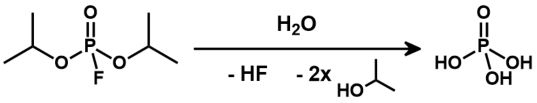

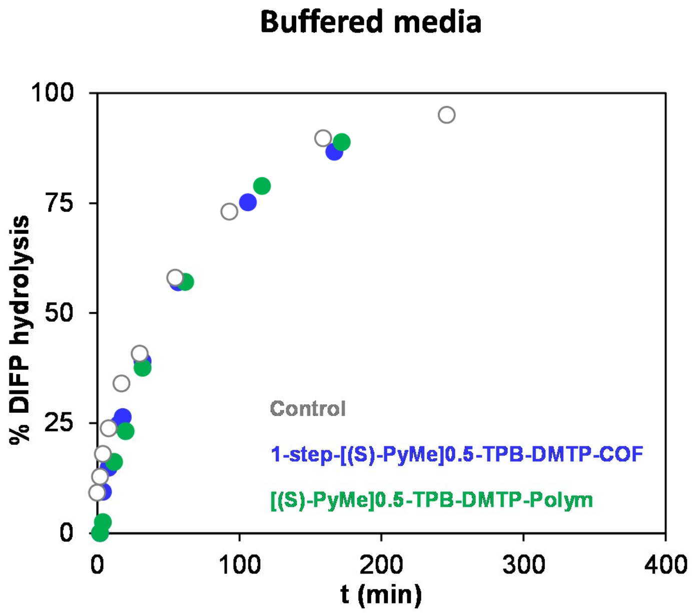

Figure S23. DIFP degradation studies in basic *N*-ethylmorpholine buffered media (pH=9.2). Reaction profiles for **1-step-[(*S*)-PyMe]$_{0.5}$-TPB-DMTP-COF** (blue) and **[(*S*)-PyMe]$_{0.5}$-TPB-DMTP-Polym** (green).

© 2019 by the authors. Licensee MDPI, Basel, Switzerland. This article is an open access article distributed under the terms and conditions of the Creative Commons Attribution (CC BY) license (http://creativecommons.org/licenses/by/4.0/).
